# The Effects of Dementia and Long-Term Care Services on the Deterioration of Care-needs Levels of the Elderly in Japan: Erratum

**DOI:** 10.1097/01.md.0000463681.24629.6a

**Published:** 2015-03-20

In the article “The Effects of Dementia and Long-Term Care Services on the Deterioration of Care-needs Levels of the Elderly in Japan,^1^ which appeared in Volume 94, Issue 7 of *Medicine*, the acknowledgments section was omitted. It should have read as follows:

The authors report no conflicts of interest. H. Lin is a recipient of a scholarship from Otsuka Toshimi Scholarship Foundation (Osaka, Japan). This study was supported by a Health and Labour Sciences Research Grant for Research on Research on Dementia from the Ministry of Health, Labour and Welfare, Japan [H26-Ninchisho-001] and JSPS Grant-in-Aid for Scientific Research (A) (25253033).
